# Swimming exercise reduces native ⍺-synuclein protein species in a transgenic *C. elegans* model of Parkinson’s disease.

**DOI:** 10.17912/micropub.biology.000413

**Published:** 2021-06-29

**Authors:** Minna Y. Schmidt, Manish Chamoli, Gordon J. Lithgow, Julie K. Andersen

**Affiliations:** 1 The Buck Institute for Research on Aging; 2 The University of Southern California, Leonard Davis School of Gerontology

## Abstract

Exercise has been historically recommended to prevent many disease conditions. Intense exercise in particular, has been shown to be beneficial for Parkinson’s disease (PD) — stopping and even reversing symptoms in some patients. Recent research in mammalian animal models of Parkinson’s have shown that exercise affects ⍺-synuclein aggregate species, considered to be a hallmark of PD. However, the exact changes in native ⍺-synuclein protein species after exercise and the downstream effects of exercise upon the health of the animals remains unclear. Recently, it was shown that swimming constitutes a form of exercise in *C. elegans* worms that confers a protective effect in several worm models of tau and Huntington protein neurodegeneration. Here we show that a period of swimming exercise (Ex) — 15-20 mins — dramatically reduces several native human ⍺-synuclein protein species in the NL5901 *C. elegans* worm model of Parkinson’s. Exercise on Day 1 of adulthood was found to improve motor function measured by the thrashing rate of worms on Day 2 and Day 4 when compared to both control (untreated) and food restricted (FR) worms. Moreover, exercised worms show smaller ⍺-synuclein::YFP puncta than food restricted worms. Here we show that exercise reduces native human ⍺-synuclein levels independent of food restriction in *C. elegans*.

**Figure 1. 15-20 mins of swimming exercise (Ex) decreases native human ⍺-synuclein protein species:**
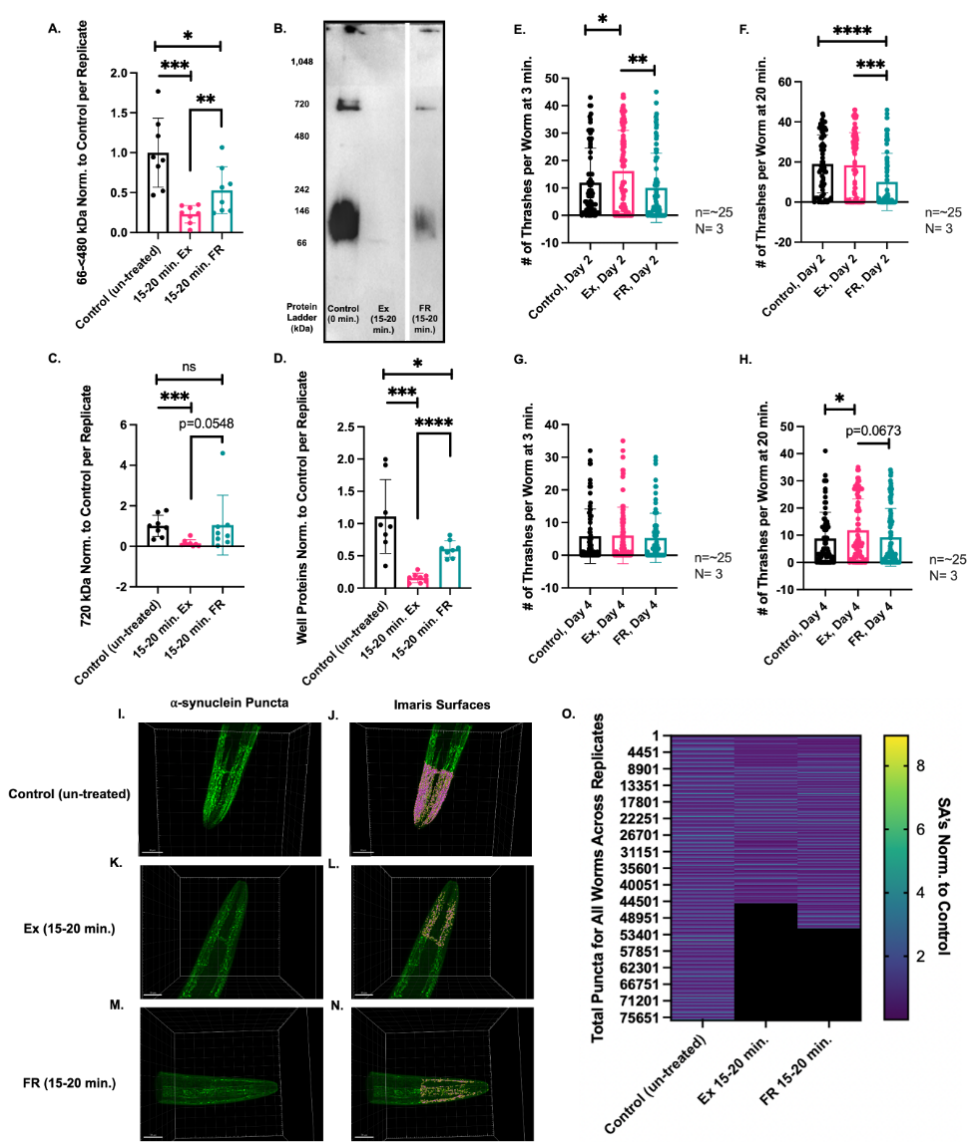
**A.** 66-<480 kDa range proteins in worms exercised for 15-20 min. on Day 1 of adulthood are significantly reduced compared to control (un-treated) and 15-20 min food restricted (FR) worms (***p≤0.0001, **p≤0.0088). These proteins are also significantly reduced for food restricted versus control (un-treated) worms (*p≤0.0116.) **B.** Representative Blue Native (BN) page western blot showing the protein profile of control (un-treated), Ex, and FR worms, demonstrating a reduction in proteins in 66-<480 kDa, 720 kDa, and well proteins. **C.** 720 kDa proteins in Ex worms are significantly reduced compared to control (un-treated) worms and almost significantly reduced compared to FR worms (***p≤0.0008, p=0.0548.) 720 kDa proteins are not significantly reduced in FR worms (p=0.4301). **D.** Well proteins in Ex worms are significantly reduced compared to control (un-treated) and FR worms (***p≤0.0002, ****p<0.0001). Well proteins are also significantly reduced in FR worms as compared to control (un-treated) worms (*p≤0.0144). N= 8, n=25 per replicate for A-D. **E.** Thrashing assay results on Day 2 show that worms previously exercised for 15-20 min on Day 1 have a significantly higher thrashing speed at 3 min compared with control (un-treated) (*p≤0.0250) or FR worms (**p≤0.0027). **F.** Thrashing assay results on Day 2 at 20 mins show that control (un-treated) worms have attained a higher thrashing rate, matching that of Ex worms and are significantly faster than FR worms (****p<0.0001). Ex worms maintain approximately the same thrashing speed as at 3 mins, which is also significantly higher than for FR worms (***p≤0.0003). FR worms do not see an increase in thrashing speed from 3 mins to 20 mins. **G.** Thrashing assay results on Day 4 demonstrate that at 3 mins, all three treatment groups display approximately the same lower thrashing speed as compared to Day 2 results. **H.** Thrashing assay results on Day 4 at 20 mins show that Ex worms have a significantly higher thrashing rate than control (un-treated) worms (*p≤0.0371) and an almost significant trend towards a higher thrashing rate compared with FR worms (p=0.0673). All worms show an improvement in thrashing rate compared to their speed at 3 mins; Ex worms show an average thrashing speed similar to that of control (un-treated) worms on Day 2. N=3, n=~25 worms per replicate for E-H. **I-N.** Representative confocal images of ⍺-synuclein puncta (green) after background subtraction was applied for **I.** control (un-treated) worms; **K.** Ex worms; and **M.** FR worms, and with calculated Imaris surfaces (pink) for **J.** control (un-treated) worms; **L.** Ex worms; **N.** FR worms. **O.** Heat map showing individual surface areas (SA’s) for each puncta for each worm across all biological replicates normalized to respective controls (right Y-axis). Total puncta count for all worms combined is shown on left Y-axis. Exercise shows a higher concentration of smaller sized puncta shown in dark blue (smaller than 2 units as seen in legend.)

## Description

Parkinson’s disease (PD) patients have been shown to benefit greatly from intense physical activity (Schenkman *et al.* 2018). Recent studies have demonstrated that exercise causes changes in the levels of ⍺-synuclein aggregate species, a hallmark of PD, in different mammalian animal models (Koo and Cho 2017; Shin *et al.* 2017; Zhou *et al.* 2017; Minakaki *et al.* 2019). However, questions still remain about how exercise affects specifically native ⍺-synuclein protein species directly after the cessation of exercise and the longer-term downstream effects which exercise may have on organismal health. It was recently discovered that periods of thrashing in liquid solution, otherwise called swimming exercise, in *C. elegans* worms, induces many mechanisms invoked during mammalian exercise (Laranjeiro *et al.* 2017). This has provided an avenue for studying exercise conditions in various *C. elegans* models of neurodegeneration (Laranjeiro *et al.* 2019). In order to study the effect of exercise on native human ⍺-synuclein protein species, we utilized the NL5901- pkIs2386 worm model of Parkinson’s which contains human ⍺-synuclein tagged to a yellow fluorescent protein (YFP) in the muscle cells (van Ham *et al.* 2008). We performed tissue analysis via Blue Native (BN) page westerns and confocal microscopy. In addition, because pharyngeal pumping is decreased while worms are swimming, we controlled for this effect by exposing worms in parallel to a period of food restriction (FR) conditions (Vidal-Gadea *et al.* 2012). We also performed thrashing assays to assess longer term downstream behavioral effects on the animals after either exercise or food restriction conditions.

It has been previously reported that NL5901 worms exhibit a lower thrashing ability than N2 worms due to the presence of human ⍺-synuclein species in the muscle cells (Anand *et al.* 2020). In our experiments, we subjected Day 1 NL5901 worms to a period of swimming exercise — 15-20 mins — and observed a dramatic effect upon the quantity of native human ⍺-synuclein proteins via BN Page westerns using an anti-GFP antibody to detect ⍺-synuclein::YFP proteins as previously described (Figure 1 A-D) (van Ham *et al.* 2008). As worms continue to swim while they are being collected for Western Blot assays, and because the speed of this collection can vary, we have indicated the 15-20 mins time frame in order to account for this variation. Three different protein groups were analyzed — 66-<480 kDa, 720 kDa, and proteins trapped within the wells (well proteins), which may be large molecular weight protein species. In the exercised worms, 66-<480 kDa and well proteins were found to be significantly decreased compared to control (un-treated) and food restricted worms. Interestingly, we also observed that the protein profile in the 66-<480 kDa range was different for exercised versus food restricted worms — while in exercised worms there appeared only a single band for ⍺-synuclein at ~90 kD, in food restricted worms we detected a range of protein similar to, but less abundant than those proteins present in control (un-treated) worms (Figure 1B). It has been shown that ⍺-synuclein (~14 kDa) runs at a higher molecular weight (~50 kDa) in BN page gels (Bartels *et al.* 2011). Therefore, we hypothesize that ⍺-synuclein tagged to YFP (~41 kDa in SDS gels) is the ~90 kDa band visible in the BN page gel in Figure 1B. For FR worms, of the three protein groups analyzed, only the 66-<480 kDa range of proteins and the well proteins show a statistically significant reduction when compared to control (un-treated) worms (Figure 1A and D, respectively), while 720 kDa proteins do not show a significant decrease (Figure 1C). It has been previously reported that exercise increases autophagy and plays a role in attenuating levels of ⍺-synuclein in an MPTP/P mouse model of Parkinson’s disease (Koo and Cho 2017). We therefore hypothesize that the decrease in human ⍺-synuclein protein species we have observed due to 15-20 min of swimming exercise in the NL5901 *C. elegans* model of PD may be attributed to protein degradation through an increase in autophagy.

We also utilized thrashing assays in order to determine the physiological effects of exercise and food restriction on ⍺-synuclein expressing worms. This method is commonly used to quantify worm neuromuscular function and is performed by quantifying the rate with which the worms swim, or thrash, during a 30 second interval. Since our results have shown that placing NL5901 worms into liquid and allowing them to thrash/swim causes ⍺-synuclein protein species to decrease, we performed the thrashing assay at 3 mins, a time point which does not induce a strong effect upon ⍺-synuclein protein species. In addition, in order to observe the effect of a full bout of swimming exercise upon ⍺-synuclein protein species in real time, we allowed worms to continue swimming undisturbed until 20 mins and recorded their thrashing rates at this time as well. On Day 2, we found that at the 3 min time point, previously exercised worms showed a significantly higher thrashing rate than control (untreated) or FR worms (Figure 1E). By 20 mins (i.e. after a period of exercise), control (untreated) worms had improved their thrashing rate and attained the speed of previously exercised worms (Figure 1F), suggesting a reduction in ⍺-synuclein aggregation due to swimming, as supported by the BN page results (Figure 1 A-D). In contrast, FR worms did not improve their thrashing speed over the 20 min period, indicating that prior food restriction may have negative effects on either ⍺-synuclein aggregation and/or the health of the worms.

Thrashing assays were also performed on Day 4. At the 3 min mark, the worms did not exhibit any differences between the three conditions, albeit all displayed lower thrashing rates than on Day 2 (Figure 1G). The phenomenon of reduced thrashing rates with age has also been previously observed in N2 worms (Hahm *et al.* 2015). After 20 mins however, previously exercised worms achieved a significantly higher thrashing rate than control (un-treated) worms and a trend towards a significantly higher rate than FR worms (p≤0.0673) (Figure 1H). In addition, previously exercised worms achieved a thrashing rate similar to the control (un-treated) worms on Day 2, suggesting that exercise exerts a restorative effect upon older NL5901 worms. We also noticed that unlike on Day 2, FR worms were able to improve their thrashing frequency between 3 mins and 20 mins, indicating that any negative effect which food restriction may have had at Day 2 was reduced by Day 4.

Using confocal microscopy, we assessed the effect of exercise and FR on the surface area (SA) size of ⍺-synuclein puncta located in the head region of the NL5901 worms as previously reported (Goya *et al.* 2020) (Figure 1 I-N). Here, we observed that exercised and FR worms have less puncta overall than control (un-treated) worms. However, we also observed that the average surface area values for the puncta in each worm did not reflect significant differences between Ex and FR conditions (calculations not shown.) Therefore, we investigated if differences may be more apparent between these conditions if instead, we observed the individual sizes of each puncta rather than the average values calculated per worm. As shown in the heat map in Figure 1O, exercised worms have a greater concentration of smaller puncta (dark blue) than control (un-treated) or FR worms. All values were normalized to their respective controls and correspond to values from 0 to 3. We hypothesize that the preparation of samples in PFA solution may have resulted in a window of time where worms continued to swim in solution, and ultimately, may have caused smaller differences across the three separate conditions, as observed in panels I-N, particularly between exercised and FR worms.

We observed that 15-20 mins of swimming exercise on Day 1 of adulthood caused a dramatic effect upon ⍺-synuclein protein quantity and species, as well as improved the thrashing rate of worms on Day 2 and Day 4 (Figure 1 A-H). Currently, we hypothesize that autophagy mechanisms may be upregulated during this brief period of exercise leading to the dramatic differences in ⍺-synuclein protein species observed. Exercise on Day 1 also allowed worms on Day 4 to reach the thrashing frequency of Day 2 control (un-treated) worms after 20 mins of swimming (Figure 1H). In contrast, FR on Day 1 appeared to hinder the swimming abilities of worms on Day 2 (Figure 1E, F.) By Day 4 however, food restricted worms appeared to be rehabilitated, although they still showed a trend towards a slower swimming pattern than exercised worms after 20 mins of swimming (Figure 1H). In support of this data, our confocal analysis shows that exercised worms have a higher concentration of smaller puncta (Figure 1O.) However, we observed that while both exercised and FR worms have less puncta than control (un-treated) worms, the differences between exercised and FR worms are not as dramatic as in the results observed in the BN page and thrashing assays. We are currently exploring differences between exercised and food restricted worms using alternate confocal methods and/or sample preparation. In the future, we are also interested in exploring further the mechanistic basis for the observed divergent effects of Ex versus FR on ⍺-synuclein aggregates. However, our results indicate the presence of a set of unique and separate relationships between ⍺-synuclein aggregation in the context of exercise versus food restriction.

## Methods

NL5901- pkIs2386 [unc-54p::alphasynuclein::YFP + unc-119(+)] worms, containing human overexpressed ⍺-synuclein protein, were purchased from CGC funded by the NIH Office of Research Infrastructure Programs (P40 OD010440).: All worms were maintained on nematode growth medium (NGM) at 20^o^ C as previously described (Brenner 1974). Worms were age synchronized by performing egg lays with ~50 worms per plate for 3 hours. Eggs were grown for 3 days to Day 1 of adulthood. Assays were performed in three separate biological replicates unless otherwise stated.

Swimming Exercise Experiments:

Three conditions — control (un-treated), exercise (Ex), and food restriction (FR) — were analyzed per biological replicate in a single day stacked by three minute intervals. Approximately 25 worms were used per condition. Briefly, Day 1 worms were placed into 1 mL S-basal solution on 10 mm un-spotted agar plates. Plates were left on the bench top undisturbed (to avoid spilling and un-intentional stimulus which may alter movement) covered with a foil leaf to block out excess light. After 15-20 mins of exercise, worms were collected into 1.5 mL Eppendorf tubes and allowed time to sink to the bottom of the tube (30 seconds to 1 min). Excess liquid was carefully removed, leaving behind approximately 30 uL of solution. Worms were immediately flash frozen in liquid nitrogen and then placed onto dry ice before storage at -80°C. For food restricted worms, Day 1 worms were picked and placed onto a small drop of S-basal solution on top of 10 mm unspotted agar plates, facilitating transfer and allowing dilution of excess bacteria present during transfer. Worms were then quickly and carefully spread with a pick onto the plate to prevent swimming. As with swimming exercise conditions, plates were left on the benchtop undisturbed for the allotted period of time to match conditions for exercised worms. After 15-20 mins (food restriction treatment times match exercise treatment times), worms were carefully picked into 30 uL of S-basal solution in a 1.5 mL Eppendorf tube and immediately flash-frozen in liquid nitrogen to prevent swimming in the test tube. Samples were stored as described above. For control (un-treated) worms, Day 1 worms were picked directly from a synchronized population plate, collected as described for food restricted worms, and stored as described above.

Blue-Native Page (BN-Page) Western Blot:

Flash frozen samples were removed from storage at -80ºC and resuspended in 10 uL of Worm Lysis Buffer (WLB) containing protease inhibitor cocktail (Roche.) Samples were thawed to room temperature and afterwards were placed on ice. Tissues were then lysed using a probe sonicator (550 Sonic Dismembrator, Fischer Scientific) at 4ºC by slowly increasing the setting dial from 1 to 3 until samples formed a white froth. Samples were then centrifuged at 13,000 rpm (centrifuge 5424, Eppendorf) for 30 seconds. Further sample preparation was performed following previously published protocols from Goya *et al.* 2020 and Thermofisher with the specification that G-250 was added at 25% of the detergent (WLB) concentration. Samples were vortexed (VWR) for ~9 seconds, spun down ~36 seconds in a hand centrifuge (VWR), and stored on ice prior to gel loading (Invitrogen.) A 40 uL volume of solution was loaded per well.

After transfer, blots were fixed in 8% acetic acid solution (glacial acetic acid, Sigma) for 15 mins and then rinsed with DI water. Blots were air dried, washed with methanol and afterwards blocked for 30 mins in 5% milk-PBST solution. Blots were incubated with 1:500 anti-GFP antibody (Rabbit polyclonal, Santa Cruz) at 4ºC overnight in a test tube rotator. The next day, blots were washed and blocked with secondary (goat anti-rabbit, Millipore) for 1 hour at room temperature and then washed again before visualization using High Sensitivity ECL solution (Chemiluminescent HRP Substrate, Millipore) on ChemiDoc MP (Biorad). A 60 second time window with 15 images was used. Protein bands were quantified using ImageJ software and statistical analysis was performed using un-paired, one-tailed t-test. Approximately 25 worms were used per condition with a total of eight biological replicates per condition.

Thrashing Assay:

Approximately 100 Day 1 worms were selected for each condition (control, exercise, or food restriction) and worms were handled similarly as described above in swimming exercise experiments. After treatments, worms were carefully transferred to separately labeled bacterial plates. On Day 2 and Day 4, 25-36 worms were picked from each bacterial plate and subjected to thrashing assays. Briefly, worms were placed into 1 mL S-basal solution in unspotted 10 mm agar plates as described above. As quickly as possible, plates were carefully carried to a microscope camera (Leica DFC 400) and recorded for 30 seconds at 3 mins and at 20 mins post-immersion in S-basal solution. Plates were not disturbed for the entirety of the assay. Thrashes were later scored from recordings. A thrashing movement was identified as when worms exhibited one entire cycle of head and tail movement. Data was analyzed using mixed effects model and un-paired, one-tailed t-test.

Confocal Microscopy:

PFA fixation: Worms were subjected to the different experimental conditions (control (un-treated), Ex, and FR) as described above. After treatment, worms were collected in 1.5 mL Eppendorf tubes in approximately 30 uL of S-basal solution as above. Worms were then immersed in PFA solution as previously described (Laranjeiro *et al.* 2019). Briefly: 4% Paraformaldehyde solution in PBS (Santa Cruz Bio) was diluted to 2% with equal volume PBS (VWR.) 1 mL of 2% PFA was quickly added to the tubes. Worms were incubated in PFA for 30 mins in foil-wrapped test tubes. Afterwards, test tubes were centrifuged in a hand centrifuge (VWR) until all worms collected at the bottom. Excess PFA was removed leaving ~30 uL. 1 mL of S-basal was added to Eppendorf tubes, and worms were stored at 4ºC.

Glass slide preparation: The day of the confocal experiment, PFA treated worms were removed from refrigeration and pipetted from Eppendorf tubes onto fresh bacterial plates. Plates were dried in a chemical hood, although over-drying of the worms was avoided. Worms were picked onto glass slides containing an agar pad (prepared as evenly as possible to avoid complications with confocal imaging i.e. Z-stack ranges) into a small drop of S-basal solution in order to facilitate transfer. After all worms were placed on agar, a small drop of Vectashield (Vector Laboratories) was pipetted on top of the worms. Solution containing the worms was gently swirled with a pick to allow unilateral mixing of Vectashield and to avoid worms overlapping. Overspreading of worms and removal from Vectashield solution was avoided. A glass coverslip was quickly affixed with nail polish to avoid drying of worms. Only one condition was prepared on a slide at a time and slides were prepared only 30 mins to 1 hour prior to visualization.

Confocal microscopy (Zeiss LSM 780 inverted confocal): Worms were visualized on slides using 10x and 20x objectives and positions fixed using the Zeiss program. Only the head region was located. The objective was then changed to 40x oil and appropriate Z-stack extremes were set. Positions were updated to reflect the new Z-plane and scanning was initiated (approximately 1 hour to 3 hours depending on number or worms and Z-stacks required (depends mainly on differences in agar pad thickness throughout slide)).

Quantification of puncta: Scans were analyzed in Imaris. Images were processed with background subtraction values of 1 um. Appropriate thresholding was set to generate surfaces for puncta. Settings were kept consistent for all conditions and biological replicates. Punta count per head region and individual puncta surface area (SA) values were analyzed. Statistical analysis was performed using 2-way ANOVA and un-paired, one-tailed t-test.
